# TAD's for the Derotation of 90° Rotated Maxillary Bicuspids

**DOI:** 10.1155/2021/4285330

**Published:** 2021-07-28

**Authors:** Hasan Sabah Hasan, Ayshan Kolemen, Mohamed Elkolaly, Anand Marya, Shreyas Gujjar, Adith Venugopal

**Affiliations:** ^1^Orthodontic Department, Khanzad Teaching Center, General Directorate of Hawler-Ministry of Health, Erbil, Iraq; ^2^Orthodontic Department, Al-Mustaqbal University College, Babel, Iraq; ^3^Orthodontic Department, Royal Dental Center, Alexandria, Egypt; ^4^Section of Orthodontics, University of Puthisastra, Phnom Penh, Cambodia; ^5^Sri Balaji Dental College, Moinabad, Telangana, India; ^6^Department of Orthodontics, Saveetha Dental College, Saveetha Institute of Medical and Technical Sciences, Saveetha University, Chennai, India

## Abstract

It is undeniable that the advent of extra-alveolar mini-implants for anchorage purposes has revolutionized the field of Orthodontics. This case report sheds light on an innovative anchorage plan using TADs, to carry out treatment for a 15-year-old female patient. The patient reported to the clinic with a chief complaint of rotated second premolars, crowding, and a deep bite. On examination, it was seen that the patient had a Class I skeletal pattern, Class II subdivision molar relationship, 90-degree maxillary second premolar rotations, crowding in both the arches, and a deep bite. In this case, the clinicians decided to use TADs for premolar derotation as it not only provides a pure rotational couple without any deleterious effects on the adjacent teeth but also helps shorten the overall treatment time. The total treatment time for this case was 10 months.

## 1. Introduction

Any tooth rotation is considered a malocclusion that requires management by an orthodontist. A rotated premolar leads to not only occlusal problems but also esthetic concerns [1, 2]. Derotation of premolars must be carried out during the initial phase of fixed orthodontic mechanotherapy [3]. Derotation of posterior teeth provides for space that can be utilized to relieve crowding in the anterior region. Derotation of the teeth, if carried out with a continuous wire, can lead to unwanted iatrogenic effects on the anchor teeth. Therefore, such rotated teeth need to be derotated using couple mechanics that involve the use of equal and opposite forces that bring about pure rotational movement [3, 4].

Patients undergoing fixed orthodontic therapy desire timely completion of their treatment which is why it is essential for an orthodontist to correct the malocclusion without any associated iatrogenic problems. This is where the use of temporary anchorage devices (TADs) has revolutionized fixed orthodontic therapy. The judicious use of TADs for treating orthodontic patients allows an orthodontist to plan for a broader range of movements [5, 6]. This case report highlights the use of TADs to treat a 15-year-old patient that reported to the clinic with a Class I skeletal pattern, a Class II subdivision molar relationship, 90-degree maxillary second premolar rotations, crowding in the maxillary and mandibular arches, and an anterior deep bite.

## 2. Case Presentation

**Figure 1:**
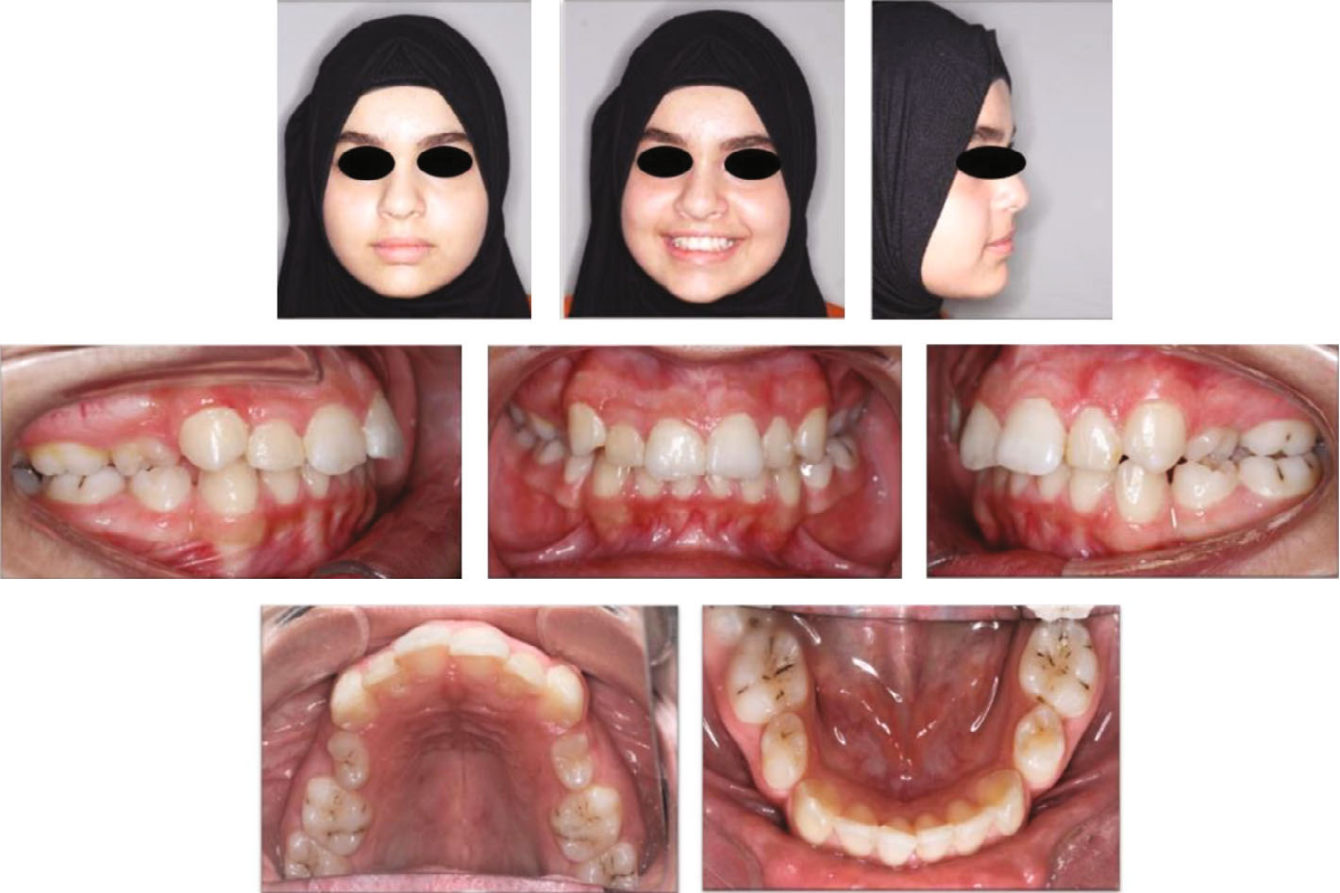
Pretreatment extra-oral and intra-oral pictures.

A 15-year-old patient reported to the clinic with a chief complaint of malaligned teeth and desired orthodontic treatment. Her previous dental history was unremarkable. On extraoral examination, she had a good facial form and proportion with a straight profile and a minimal display of buccal corridors on smiling. Intraoral examination showed mild crowding in both the maxillary and the mandibular arches, a Class I molar relation on the right side, and a half-unit Class II relation on the left side. In addition, the canine relationships were half-unit Class II on both sides, the overjet was normal, there was a 4 mm anterior deep bite, there were missing upper and lower right/left first premolars due to previous extractions, and both the maxillary second premolars were present with 90-degree rotations (Figure 1).

**Figure 2:**
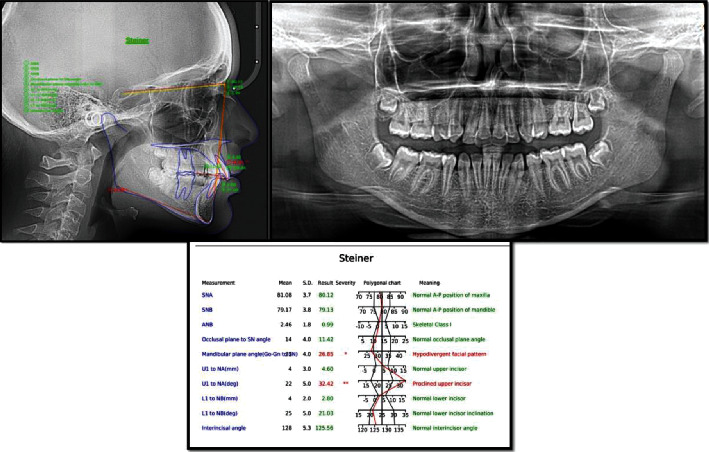
Pretreatment cephalogram and panoramic radiograph with analysis of the lateral cephalogram using the Steiner analysis.

The panoramic radiograph showed no abnormality except for missing upper and lower, right/left first premolars. Cephalometric analysis was carried out using WebCeph software (AssembleCircle Corp., Republic of Korea) and revealed a skeletal Class I relationship, a hypodivergent facial pattern, and proclined upper incisors (Figure 2).

## 3. Treatment Objectives

1. Develop a unique anchorage design using TADs for derotation of the maxillary second premolars without compromising the anchorage units
2. Apply a pure rotational couple force without effect on the adjacent teeth
3. Reduce the overall treatment time
4. Alleviate crowding in both the maxilla and the mandible
5. Correct the anterior deep bite
6. Correct the molar and canine relationships

## 4. Treatment Planning

The patient showed a Class I skeletal base, CI II subdivision molar relationship, mild crowding, deep bite, hypodivergent facial pattern, procline upper incisors, and missing upper and lower right/left first premolars and both upper second premolars with 90-degree rotation.

The treatment plan was proposed as follows:

1. Leveling and alignment of the maxillary and mandibular arches using fixed orthodontic therapy
2. Placement of two mini-implants, one in the buccal aspect and the other in the hard palate for derotation of rotated premolars simultaneously while leveling and aligning the arch in order to save time
3. Closure of residual spaces
4. After treatment completion, retention with the upper and lowered fixed bonded lingual retainers

## 5. Treatment Alternatives

Three alternatives were proposed to achieve treatment goals:

1. The first option was to use a stiff.019 x.025^″^ SS base arch wire after leveling and alignment in the maxillary arch. Reinforcing the anterior anchorage from the canine to canine (joining the segment using tight ligature ties) as well as a posterior anchorage (by joining first and second molars together using a ligated wire). These anchorage units would be used to derotate the premolars using power chains from the buccal surface of the second premolars to the hook of the molar tube and from the lingual surface of the second premolars to the lingual surface of the upper canines (using lingual buttons). Bite turbos would be placed to disocclude the premolars during the alignment phase
2. The second option was to again use a stiff.019 x.025^″^ SS wire as the base arch wire. The anterior anchorage would be the same as the first option, but in this case, the posterior anchorage would be reinforced using a Transpalatal arch bonded to the first maxillary molars. The derotation would be carried out using similar biomechanics to the first option
3. The third option was to use TADs as maximum anchorage so that derotation of the premolars could be carried out at the same time as the leveling and alignment of the other teeth. Four TADs would be placed between the upper first molars and second premolars buccally, and two would be placed between the upper canine and second premolars on the palatal aspect in the hard palate without using bite turbos. The TADs would be utilized to derotate using couple mechanics and maintain an intrusive effect on the premolars

After careful consideration of all the three treatment options, the third option was chosen as it shortened the treatment time and helped achieve the derotation of the premolars without any adverse effects on the adjacent teeth.

## 6. Treatment Progress

Prior to bracket placement, impressions were taken for fabrication of study models, followed by oral prophylaxis. Fixed orthodontic appliance was bonded (slot 0.022-inch, Roth prescription, Discovery, Dentaurum, Germany) with four lingual buttons (Dentaurum, Germany) on the buccal and lingual aspects of second premolars. At the same time, four TADs (used as anchorage for derotation of the first premolars; 1.6 × 8 mm) (Gssem, Korea) were inserted: the first two were inserted in the left/right buccal aspects between the upper second premolars and first molars, and the other two were inserted in the hard palate between the canines and second premolars. Both TADs were positioned close to the occlusal surface to reduce the intrusion effect on premolars.

**Figure 3:**
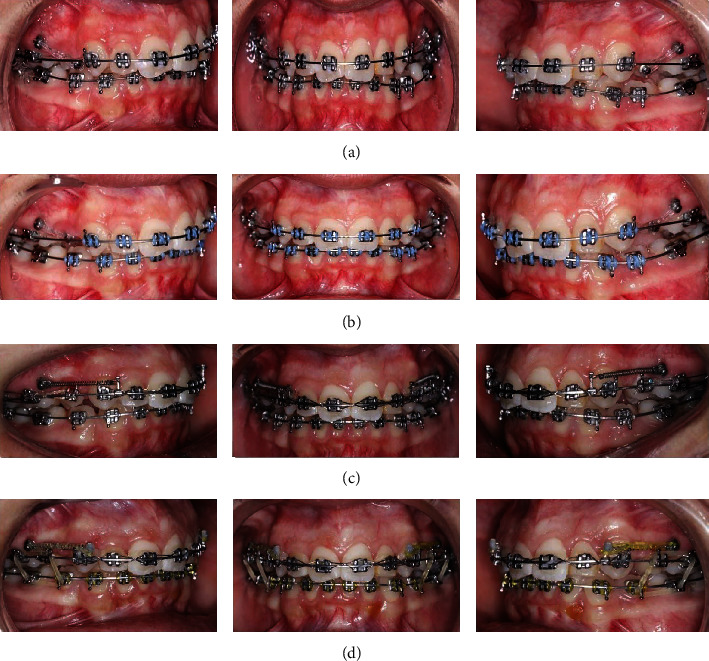
Showing treatment progress; (a, b) simultaneous leveling and alignment stage with derotation of the upper second premolars, (c) en masse retraction, (d) usage of elastic rings for maximum intercuspation.

**Figure 4:**
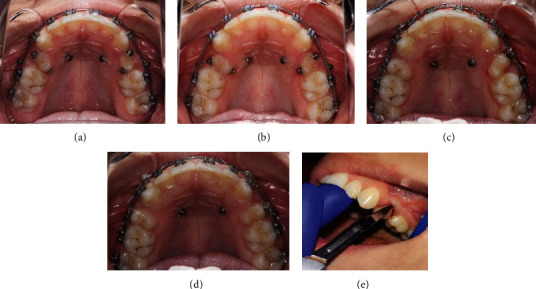
(a–c) Occlusal pictures showing derotation of premolars using couple mechanics. (d) Space closure. (e) CSF technique.

Leveling and alignment was started on a 0.014^″^ NiTi archwire and followed by a sequence of 0.016^″^, 0.018^″^, and 0.016 × 22^″^NiTi along with active power chains (clear continuous; Vimel, Koreas, changed every two weeks) from the TADs to the attached buttons on the second premolars. The elastic chains were placed to derotate the premolars using pure couple forces [7] (Figures 3(a), 3(b), 4(a), and 4(b)).

After completion of the derotation, the buttons were removed and brackets were bonded on the second premolars. A.016 x.022^″^ NiTi wire was placed in the bracket slots of the derotated premolars to align them. Once we were able to engage a.019 x.025^″^ SS wire in the slot, the palatal TADs were removed and the buccal screws were used as anchorage. Closure of remaining spaces was done with hooks placed between the canines and the lateral incisors and placement of 6 mm closed coil springs from the buccal TAD to the hook. The residual space left after premolar alignment was eventually closed and was helpful in the correction of the anterior deep bite. Additionally, inter-arch elastics were placed between the maxillary and mandibular premolars and first molars (1/4-inch, medium, Dentaurum, Germany) to settle the occlusion. (Figures 3(c), 3(d), 4(c), and 4(d)) Once the occlusion was well settled, the fixed appliance was debonded using a Carbide bur (TDF-6R, Brassler, USA) and retention was provided. Circumferential supracrestal fiberotomy (CSF) was performed on the second premolars to avoid any further chances of relapse (Figure 4(e)).

## 7. Results

**Figure 5:**
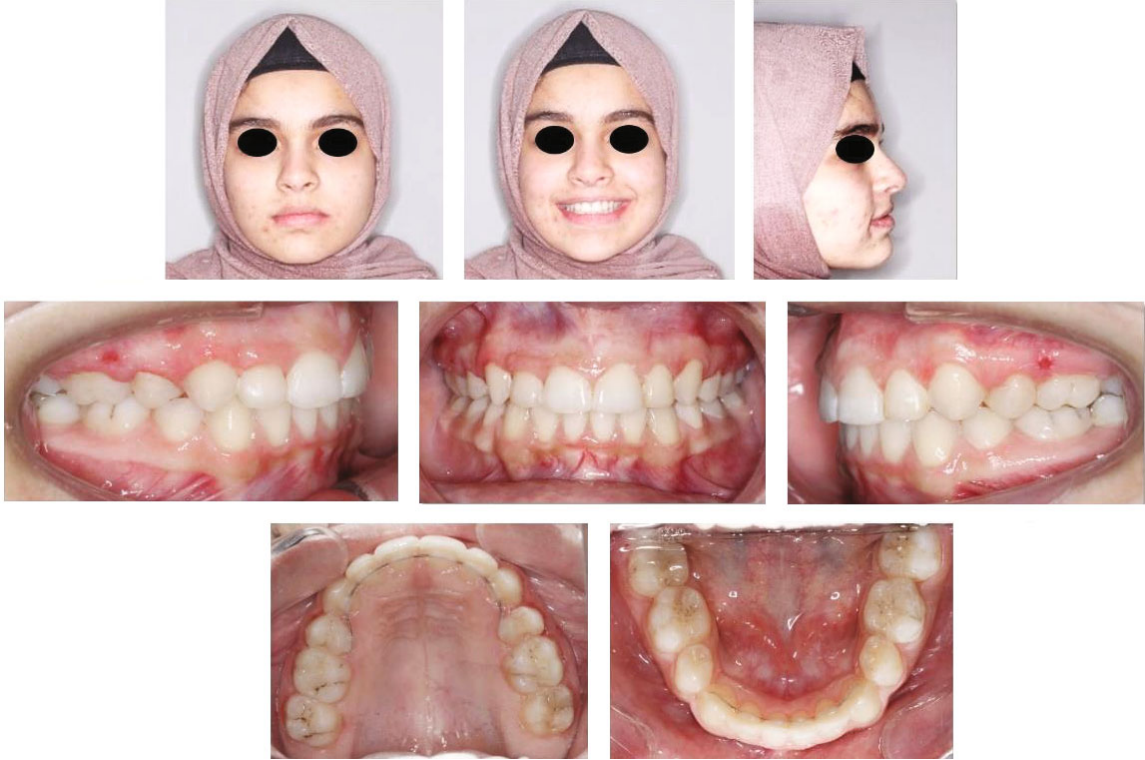
Posttreatment extra and intraoral images.

**Figure 6:**
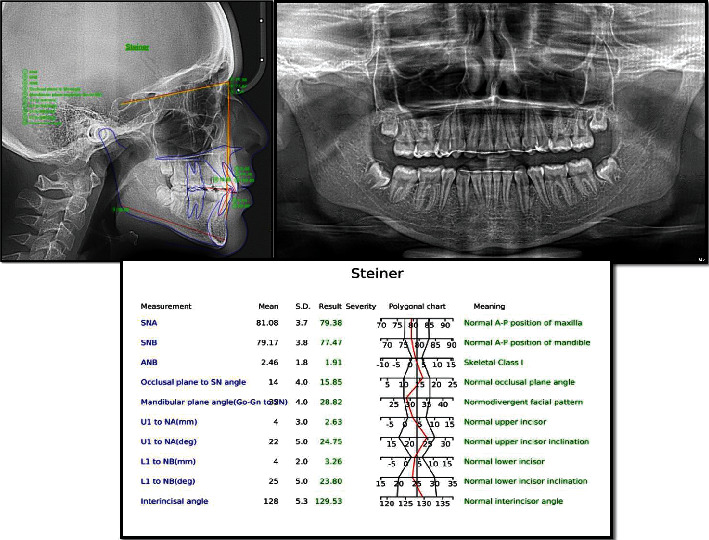
Posttreatment cephalogram and panoramic radiograph with analysis of the lateral cephalogram using the Steiner analysis.

**Figure 7:**
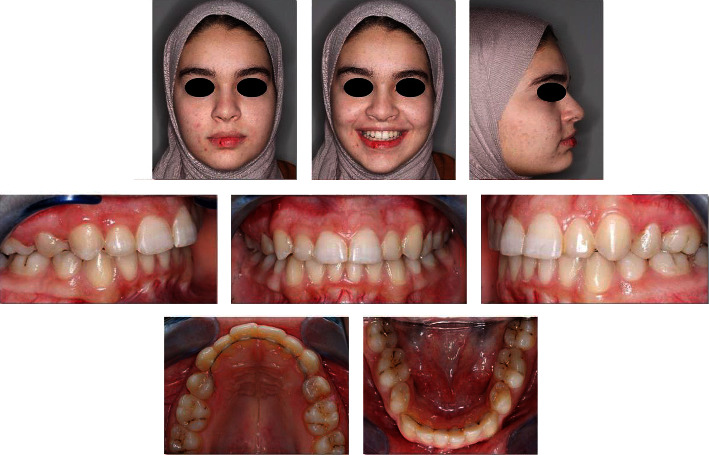
Posttreatment extra- and intraoral images 5 years posttreatment.

The orthodontic treatment was completed in ten months. Class I molar and canine relationships were obtained on both sides with ideal overbite, overjet, correct incisor inclinations, and well-aligned teeth (Figures 5 and 6). The upper and lower midlines were minimally off at the end of treatment (Figure 5). Upper right and left second premolar rotations were corrected by a customized TAD based approach. Circumferential supracrestal fiberotomy (CSF) was performed on the derotated premolars to reduce the chances of relapse. The result was retained using a fixed lingual bonded retainer using a braided stainless steel wire on the upper and lower arches (Figure 5). Additionally, clear Essex retainers were provided to the patient for full time wear. The posttreatment records demonstrate the completed case findings and follow-up after 5 years of treatment (Figure 7).

## 8. Discussion

Amongst the various treatment alternatives that have been suggested in this case report, the use of couple mechanics on adjacent teeth is most commonly preferred method. Unfortunately, due to poor control on the adjacent teeth, such mechanics lead to iatrogenic effects causing further delay in treatment.

Since TADs are being used as anchorage in clinical orthodontic treatment widely, there has been a change in treatment approach over the last few years [8–14]. Controlling anchorage in certain cases that demand strict anchorage requirements helps to avoid undesirable tooth movements. However, even a small reactive force can cause undesirable movements and so; it is important to have absolute anchorage to avoid them [15, 16]. Absolute or infinite anchorage is defined as zero anchorage loss due to the forces applied to move teeth [17]. It can only be obtained by using dental implants as anchors, relying on bone to inhibit movement. Anchorage provided by devices, such as implants or TADs fixed to bone, may be obtained by enhancing the reactive unit's support (indirect anchorage) or by fixing the anchor units (direct anchorage), thus facilitating skeletal anchorage. Orthodontic anchorage is an important factor in obtaining good treatment results. Stable anchorage is a prerequisite for orthodontic treatment with fixed appliances and especially for derotation of rotated posterior teeth [18].

A *couple* is a form of moment. It is created by a pair of forces with equal magnitudes but in opposite directions with a noncoincidental line of action. Because the forces have the same magnitude but are oppositely directed, this special force system's net potential to translate the body on which it acts is nil and there is only pure rotation. The magnitude of the couple's moment (MC) depends on both force magnitude and distance between the two forces. The moment created by a couple is the sum of the moments created by each of the two forces. If there are two forces of a couple system acting on opposite sides of the Cres, their effect to create a moment is additive. If they are on the same side of the Cres, they are subtractive [19]. Both TADs were placed in proximity to the occlusal surface reducing the intrusion on second premolars during derotation, which also allowed for disocclusion in the premolar region. The forces originating from bracket-wire interactions are transmitted to tooth structures causing displacement (unwanted tipping movement), in this case the TADs. The TADs not only provide anchorage but also help bring about pure rotational movement and minimizing the overall treatment time.

Successful orthodontic treatment depends a lot on the various strategies that are employed to prevent relapse of orthodontically treated teeth. There are many factors that may contribute to relapse of a successfully treated malocclusion. Growth in an unfavorable manner or tissue rebound may be some of the factors responsible [20]. Rotated teeth are known to relapse more commonly, and the role of the periodontium has been extensively studied in this regard [20, 21]. Reorientation and lack of reorganization of the supracrestal fibers are considered the main culprit for such relapse [22]. A surgical technique to sever these periodontal fibers was reported by Edwards [23] and was termed circumferential supracrestal fiberotomy (CSF). The theory behind this was that such a surgery would relieve the tension on the supra-alveolar fibers after derotating a tooth, reducing the chances of relapse [21]. Even with long-term retention using fixed retainers, these sets of fibers have poor reorientation or reorganizational capacity [24–26]. Many further studies have validated the efficacy of CSF as a standard to retain derotations in conjunction to other retentive measures [26–28].

Some degree of relapse appeared on upper right central incisor after five years. However, this corresponded with what Al-Jasser et al. [29]; Naraghi et al. [30] found, as they mentioned, that minor relapse in short-term follow-up (1 year) was noted in the maxillary anterior teeth after correction of irregularities compared to a 2 to 4-year period of bonded retention. This result could be attributed to failures that may occur during the retention period without any follow-up checkup of the patient. Other contributing factors to relapse are unfavorable skeletal growth as well as contact point discrepancy relative to the dental arch during the retention period. Changes in the arch form, which frequently occur during both the treatment and posttreatment periods, have also shown to influence retention [20].

Clear aligners (CAT) could also have been explored as one of the options for treating this case. However, several studies have demonstrated that auxiliaries such as attachments and elastics are mandatory in CAT (clear aligner orthodontic therapy) to achieve predictable results. Tooth shape has been shown to influence the efficiency of CAT [31–33], during correction of misalignment due to the geometric interaction between teeth and aligners. Rotation of round-shaped teeth remains one of the less predictable movements in CAT. According to the existing literature [34, 35], the least accurate movement during CAT is premolar rotation. The reduced control of orthodontic tooth movement could be related to the lack of interproximal undercuts between premolars, producing an incorrect force distribution. The biomechanical benefits of a TAD-based anchorage system such as the one used in this study can be utilized for correcting premolar rotations with aligner therapy too. A further study needs to be conducted, and more cases need to be published in orthodontic literature concerning TADs and aligners in conjunction, to understand the possibilities of widening the horizons of orthodontic treatment.

TADs may seem like an overkill to derotate severely rotated teeth, but in fact, they are the most fail-safe mechanics that can guarantee no deleterious effects on adjacent teeth, if used with proper training and understanding of orthodontic biomechanics.

## 9. Conclusion

Severely rotated teeth need a specifically designed treatment plan when using a fixed orthodontic appliance. Using TADs for correction, such rotated second premolars have a variety of benefits:

1. Provide maximum anchorage without any effect on the adjacent teeth (zero anchorage loss)
2. Help reduce the overall treatment time, as the derotations were being carried out simultaneously alongside leveling and alignment
3. Use TADs as anchorage allowed for use of pure couple forces without any deleterious side effects

## Data Availability

The data that support the findings of this study are available from the corresponding author upon reasonable request.
